# Salivary Effector Sm9723 of Grain Aphid *Sitobion miscanthi* Suppresses Plant Defense and Is Essential for Aphid Survival on Wheat

**DOI:** 10.3390/ijms23136909

**Published:** 2022-06-21

**Authors:** Yong Zhang, Xiaobei Liu, Yu Fu, Leonardo Crespo-Herrera, Huan Liu, Qian Wang, Yumeng Zhang, Julian Chen

**Affiliations:** 1 State Key Laboratory for Biology of Plant Diseases and Insect Pests, Institute of Plant Protection, Chinese Academy of Agricultural Sciences, Beijing 100193, China; zhangyong02@caas.cn (Y.Z.); xiaobeiliu7@163.com (X.L.); fuyufight@163.com (Y.F.); m15210608845@163.com (H.L.); wangqian_711@126.com (Q.W.); zhangyumemg1017@163.com (Y.Z.); 2 International Maize and Wheat Improvement Center (CIMMYT), Texcoco 56130, Mexico; l.crespo@cgiar.org; 3 Department of Entomology, College of Plant Protection, China Agricultural University, Beijing 100193, China; 4 College of Plant Health and Medicine, Qingdao Agricultural University, Qingdao 266109, China

**Keywords:** cereal aphid, salivary protein, plant immunity, RNA interference, aphid performance

## Abstract

Aphid salivary effectors play important roles in modulating plant defense responses. The grain aphid *Sitobion miscanthi* is one of the most economically important cereal aphids worldwide. However, little information is available on the identification and functional analysis of salivary effectors of *S. miscanthi*. In this study, a candidate salivary effector Sm9723 was identified, which was specifically expressed in aphid salivary glands and highly induced during the aphid feeding phase. Transient overexpression of Sm9723 in *Nicotiana benthamiana* suppressed BAX and INF1-induced cell death. Further, Sm9723 overexpression inhibited *N. benthamiana* defense responses by reducing pattern-triggered immunity associated callose deposition and expression levels of jasmonic and salicylic acid-associated defense genes. In addition, the salivary effector *Sm9723* of *S. miscanthi* was effectively silenced through nanocarrier-mediated dsRNA delivery system. After silencing *Sm9723*, fecundity and survival of *S. miscanthi* decreased significantly, and the aphid feeding behavior was also negatively affected. These results suggest salivary effector Sm9723 is involved in suppressing plant immunity and is essential in enabling aphid virulence, which could be applied as potential target gene for RNAi-mediated pest control of *S. miscanthi*.

## 1. Introduction

Aphids are one of the most economically important agricultural and forest pests because they directly draw phloem sap from sieve elements and vector various phytoviruses [1,2]. Prior to feeding, aphid stylets must penetrate the plant epidermis and mainly follow an intercellular pathway toward their feeding sites in sieve elements [3]. During probing and feeding, aphids secret two types of saliva: gelling saliva that hardens to form a sheath around the stylets, which may form a protective function, and watery saliva containing a complex mix of proteins that can be injected into the plant vascular system [4,5,6,7,8]. Aphid saliva performs important roles in modulating aphid-plant interactions [9,10,11]. Recent evidence suggests that aphids, like plant pathogens, present conserved molecules in saliva similar to pathogen-associated molecular patterns (PAMP), which are perceived by pattern-recognition receptors (PRRs) on the plant cell membrane and trigger PAMP-triggered immunity (PTI). Aphids then secrete effectors to suppress this and other types of plant defenses, thus promoting effector-triggered susceptibility. Nevertheless, these effectors deployed to suppress host defenses are recognized by plant resistance (R) proteins, activating a strong immune response, namely an effector-triggered immunity response, typically including a hypersensitive response [12,13,14].

Studies have demonstrated the capacity for aphid salivary components to induce plant defenses, whereby infiltration of watery saliva (3–10 kDa) of the green peach aphid *Myzus persicae* resulted in reduced aphid performance and upregulated expression of genes involved in plant defense signal pathways [15]. Similarly, transient expression of *M. persicae* salivary proteins, Mp10, Mp42, Mp56, Mp57, and Mp58, in tobacco *Nicotiana benthamiana* resulted in reduced aphid fecundity, highlighting their roles in inducing aphid resistance [16,17]. Armet overexpression in the pea aphid *Acyrthosiphon pisum* and infiltration of purified Armet protein in *N. benthamiana* induced salicylic acid-mediated defense in plants, enhancing plant resistance against the bacterial pathogen *Pseudomonas syringae*, but not aphids [18]. However, to date, little is known about the role of PAMP-like molecules from aphids in plant–aphid interactions.

Aphid saliva also contains several effectors that suppress plant defense responses and enhance plant susceptibility during infestation. For example, C002 is an initial effector described in *A. pisum* essential for aphid continuous phloem feeding [19,20]. Transit MpC002 overexpression in *M. persicae* and Me10, Me23, and Me47 of the potato aphid *Macrosiphum euphorbiae* in *N. benthamiana* enhanced aphid fecundity, suggesting these effectors could contribute to suppressing plant defense [16,21,22,23]. Recent studies show Me10 contributes to defense against non-adapted aphids, interacting with the tomato 14-3-3 isoform (TFT7) [24]. Expression of candidate effectors RpC002 and Rp1 from the bird cherry-oat aphid *Rhopalosiphum padi* in transgenic barley lines enhanced plant susceptibility to aphids by suppressing plant defense responses [25].

The grain aphid *Sitobion* *miscanthi* is a globally distributed sap-sucking specialist of cereals and the dominant species in wheat-growing regions across China, which has previously been misreported as *Sitobion avenae* [26]. *S. miscanthi* can induce huge yield losses by directly drawing phloem sap and transmitting various plant viruses, such as barley yellow dwarf virus [1,27]. Infestation of *S. miscanthi* can upregulate expression levels of many defense response genes in wheat [28,29] and the watery saliva secreted by *S. miscanthi* can induce aphid resistance in wheat [30]. In addition, transcriptomic analysis of *S. miscanthi* salivary glands identified 526 transcripts predicted to encode secretory proteins and 114 salivary proteins were identified in the watery saliva of *S. miscanthi* using tandem mass spectrometry [31,32]. However, few studies can identify the role of these salivary proteins in interactions between *S.* *miscanthi* and wheat. Here, a salivary gland specifically expressing salivary protein Sm9723 of *S.* *miscanthi* was selected as a candidate effector. The aims of this study were (i) to detect the effects of transient overexpressing Sm9723 in *N. benthamiana* on plant immune responses using agroinfiltration; (ii) to investigate the impact of *Sm9723* silencing via nanocarrier-mediated RNAi on *S. miscanthi* performance and feeding behavior; and (iii) to identify the crucial roles of Sm9723 in prompting aphid fitness on wheat.

## 2. Results

### 2.1. Sequence Analysis of Candidate Salivary Effector Sm9723

**Figure 1:**
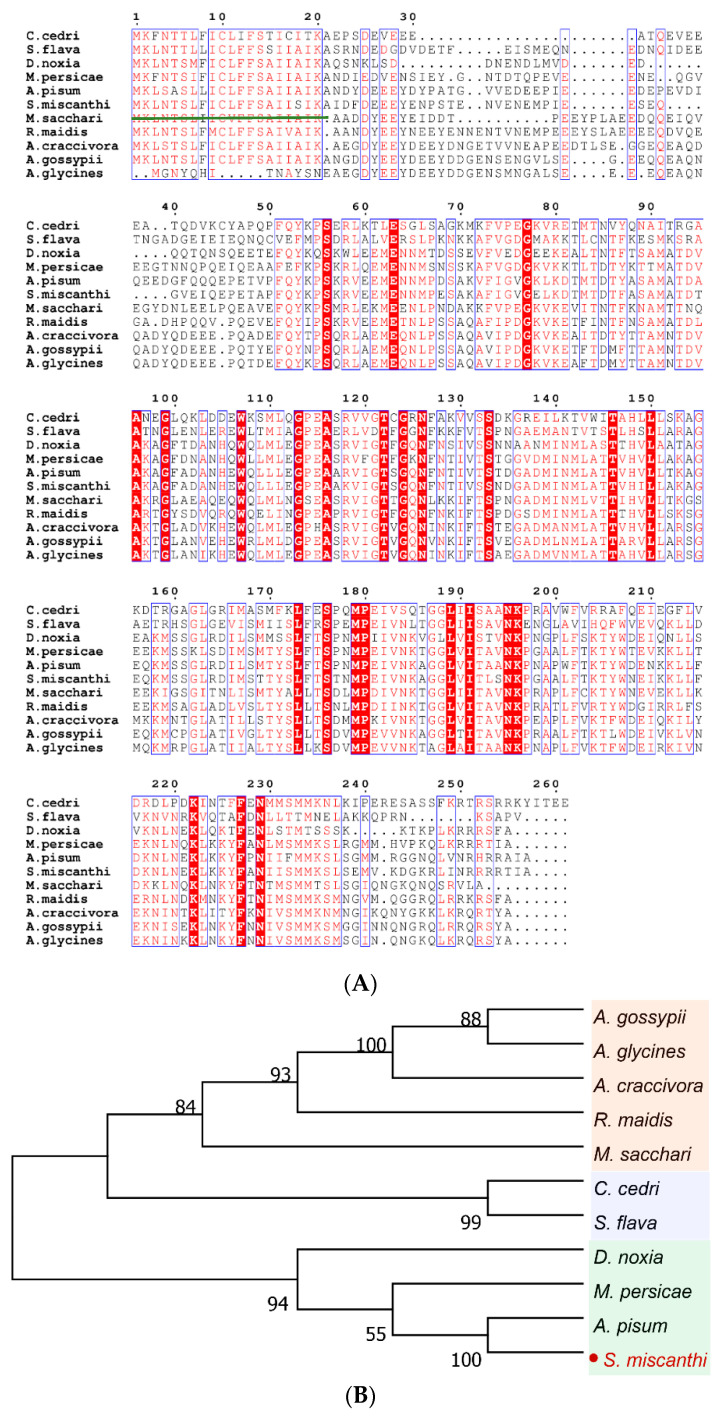
Sequence analysis of Sm9723. (**A**) Multiple sequence alignment of Sm9723 protein and orthologs from other aphid species. The deduced amino acid sequences from ten aphid species include *Acyrthosiphon pisum* (NP001313555.1), *Myzus persicae* (XP022166918.1), *Diuraphis* *noxia* (XP015374910.1), *Aphis gossypii* (XP027837168.1), *Aphis glycines* (KAE9543457.1), *Melanaphis* *sacchari* (XP025198376.1), *Rhopalosiphum* *maidis* (XP026816888.1). *Aphis craccivora* (KAF0762417.1), *Cinara cedri* (VVC37537.1), and *Sipha flava* (XP025418293.1). Red shades indicate identical amino acids. Red fonts indicate similar amino acid, and blue boxes include the sequences with identical and similar residues. Signal peptide of Sm9723 is highlighted with green underline. (**B**) Phylogenetic tree constructed by comparing the amino-acid sequences of Sm9723 and orthologs from other aphid species.

The full-length cDNA for the *Sm9723* gene contains a 798 bp open reading frame and encodes a polypeptide of 265 amino acid residues (Figure 1A) (GenBank accession number: ON783485). The first 21 amino acids constitute the signal peptide, with cleavage predicted between residues 21 and 22 (Figure 1A).

BLAST analyses revealed that Sm9723 has no strong matches to any protein of known function or to any predicted protein outside of the family Aphididae. The amino acid sequence analysis showed Sm9723 shares the highest identity (77.74%) with the *A. pisum* ortholog but only 38.49% sequence identity with *Cinara cedri* ortholog, respectively. Phylogenetic analysis showed that Sm9723 and the *A. pisum* ortholog were closely related, clustering into an independent clade (Figure 1B).

### 2.2. Expression Levels of Candidate Effector Sm9723 of S. miscanthi in Different Tissues and Various Aphid Feeding Time Points on Wheat

**Figure 2:**
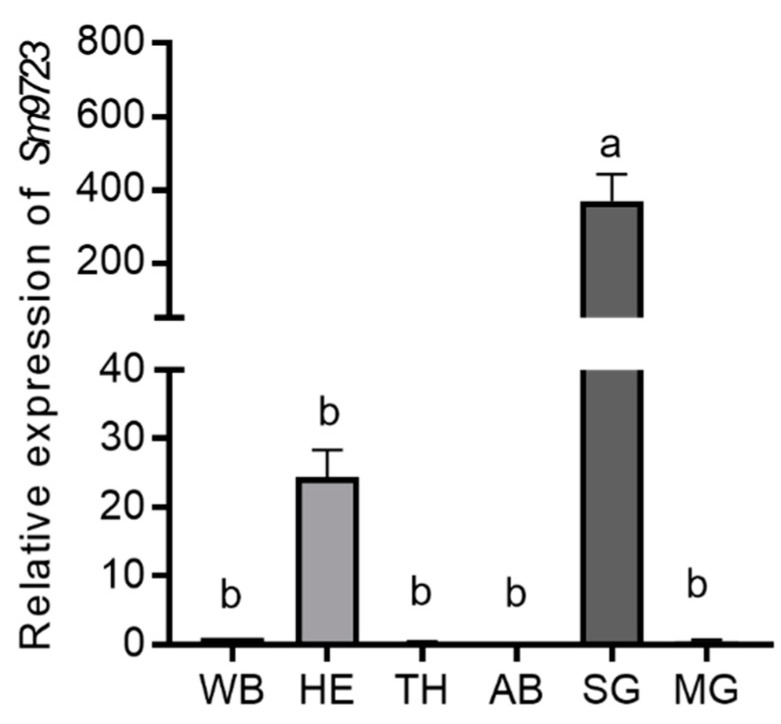
RT-qPCR results of the relative expression of *Sm9723* in different tissues of *Sitobion miscanthi*. Abbreviation for tissues: whole body of apterous adults (WB), heads (HE), thorax (TH), abdomen (AB), salivary glands (SG), and midguts (MG). *β-actin* and *NADH hydrogenase* were used as internal reference genes. Standard error (SE) is represented by the error bar. Different lower-case letters above each bar indicate significant differences among groups (one-way ANOVA followed by Duncan’s multiple range tests, *p* < 0.05).

*Sm9723* expression in different aphid tissues, including salivary glands, heads, thoraxes, abdomens, and midguts of apterous aphids, was detected using RT–qPCR. As shown in Figure 2, relative expression of *Sm9723* in the salivary gland was 370.12 ± 71.99 times higher than the whole body and was significantly higher than other aphid tissues, indicating this gene was specifically expressed in the *S. miscanthi* salivary gland.

**Figure 3:**
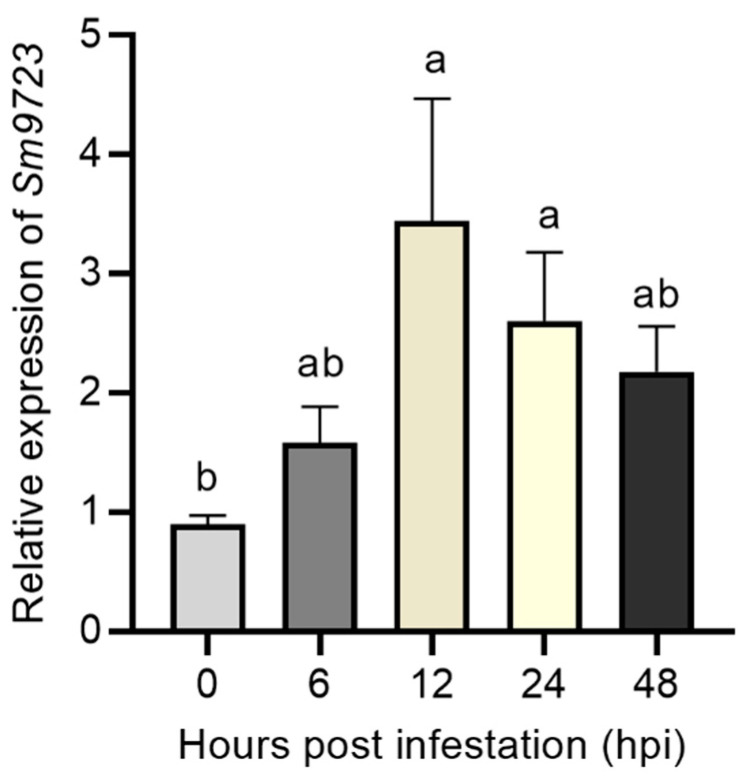
Relative expression levels of *Sm9723* gene of *Sitobion miscanthi* after infestation on wheat plants at different time points. Data are presented as mean ± SE (n = 3). Different lower-case letters above bars indicate significant differences among groups (one-way ANOVA followed by Duncan’s multiple range tests, *p* < 0.05).

To further characterize changes of transcript profiles of *Sm9723* at different aphid feeding time points, transcript levels of *Sm9723* were analyzed using RT–qPCR. The results in Figure 3 showed that expression levels of *Sm9723* were significantly induced during infestation of wheat host plants and peaked at 12 hpi with an approximately 3.44-fold increase. These results suggest *Sm9723* was significantly induced during aphid feeding and may contribute significantly to aphid virulence.

### 2.3. Overexpression of Sm9723 in Nicotiana benthamiana Suppresses Cell Death

**Figure 4:**
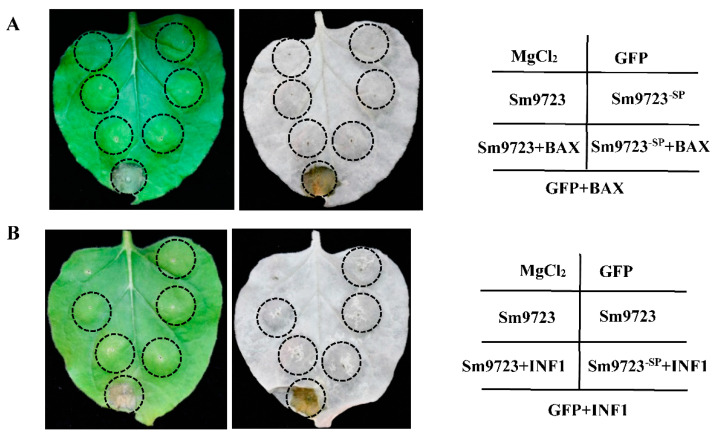
Transient overexpression of Sm9723 and Sm9723^-SP^ in Nicotiana benthamiana inhibited PCD triggered by BAX (**A**) and PAMP-INF1 (**B**). Leaves were infiltrated with MgCl_2_ and recombinant strains of A. tumefaciens cells carrying GFP were set as blank and negative control groups, respectively. Leaves were decolorized with ethanol and photographed five days after infiltration. Three biological replications were performed.

We examined the potential roles of Sm9723 in suppressing BAX and PAMP INF1-induced programmed cell death (PCD) in N. benthamiana. As shown in Figure 4, transient overexpression of both BAX and INF1 in tobacco activated obvious PCD, but overexpression of GFP, Sm9723 and Sm9723^-SP^ did not induce PCD, whereby overexpression of Sm9723 and Sm9723^-SP^ could significantly inhibit BAX and INF1-induced PCD in N. benthamiana. As a negative control, GFP overexpression did not influence PCD symptoms.

### 2.4. Transient Overexpression of Sm9723 in N. benthamiana Suppresses PTI-Associated Callose Deposition and Plant Defense Responses

**Figure 5:**
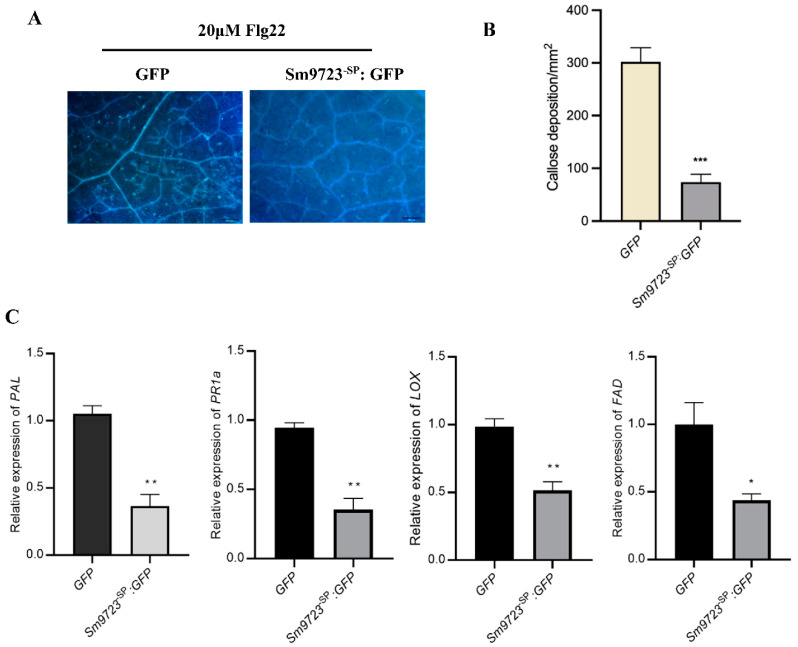
Sm9723 suppressed Flg22 induced PTI in *Nicotiana benthamiana*. (**A**) Callose deposition induced by 20 μM Flg22 in *N. benthamiana* leaves expressing Sm9723^-SP^: GFP or GFP (control). Images were taken 12 h after infiltration with Flg22. Bar = 200 μm. (**B**) Number of callose deposits in Sm9723^-SP^: GFP- or GFP-overexpressed *N. benthamiana* leaves (1 mm^2^). Data are presented as mean ± SE. Fifteen biological replicates were performed for each treatment. Asterisks above bars indicate significant differences between controls and treatments (*** *p* *<* 0.001; Student’s *t*-test). (**C**) Relative expression levels of SA responsive genes *PAL*, *PR1a*, and JA-related genes LOX and *FAD* in *N. benthamiana* leaves transiently expressing Sm9723^-SP^: GFP or GFP after infiltration with Flg22 were examined using RT-qPCR. Data are presented as mean ± SE. Three biological replicates were performed for each treatment. Asterisks above bars indicate significant differences between controls and treatments (* *p* < 0.05; ** *p* < 0.01; Student’s *t*-test).

For PTI suppression assays with *N. benthamiana*, the GFP and the Sm9723^-SP^: GFP construct were transiently expressed in leaves by agroinfiltration followed by injection with 20 μM Flg22 at 48 hpi. As shown in Figure 5A, aniline blue staining showed less callose accumulation in *N. benthamiana* leaves expressing Sm9723^-SP^: GFP than in leaves expressing GFP at 12h post infiltration of Flg22. Further, the number of callose deposits (per mm^2^) induced by Flg22 in leaves expressing Sm9723^-SP^: GFP was significantly less than control groups (Figure 5B).

At 12h post infiltration of Flg22, expression levels of jasmonic acid and salicylic acid-related defense genes, such as *PAL*, *PR1a*, *LOX* and *WRKY12* in Sm9723^-SP^: GFP-overexpressed leaves were significantly lower (0.35–0.51-fold change) as compared to control leaves expressing GFP (Figure 5C). These results indicate that Sm9723 can suppress callose deposition and defense responses in *N. benthamiana*.

### 2.5. Silencing of Sm9723 by a Nanocarrier-Mediated dsRNA Delivery System

**Figure 6:**
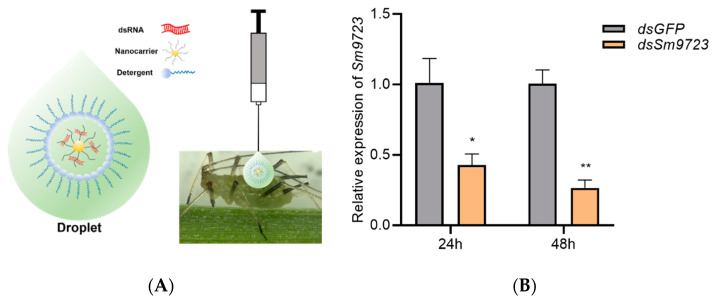
Transcript level of *Sm9723* was effectively inhibited using nanocarrier-mediated RNAi. (**A**) Schematic diagram for applying the nanocarrier-mediated transdermal dsRNA delivery system. The dsRNA/nanocarrier/detergent droplet was dropped on the notum of *Sitobion miscanthi* using microinjector. (**B**) Relative expression levels of *Sm9723* at 24 and 48 h after *dsSm9723*/nanocarrier/detergent or *dsGFP/*nanocarrier/detergent (control) treatments. Three biological replicates were conducted for each treatment (n = 3, Student’s *t*-test). The data shown are mean ± SE. Asterisks above bars indicate significant differences between controls and treatments (* *p* < 0.05; ** *p* < 0.01).

A schematic diagram of the transdermal dsRNA delivery system with nanocarriers is shown in Figure 6A. Here, 0.1 μL dsRNA formulation with 200 ng/µL *dsSm9723* was dropped onto the aphid abdomen. RNAi efficiency of nanocarrier-mediated RNAi on the *Sm9723* gene was then detected using RT–qPCR. As shown in Figure 6B, expression levels of *Sm9723* in *S. miscanthi* decreased (0.42 ± 0.14-fold change) significantly following treatment with *dsSm9723* for 24 h compared with controls. After 48 h of treatment, the transcript levels of *Sm9723* were further reduced to 0.27 ± 0.097-fold, which were significantly lower than controls.

### 2.6. Effects of Sm9723 Silencing on Fecundity and Survival Rate of S. miscanthi

**Figure 7:**
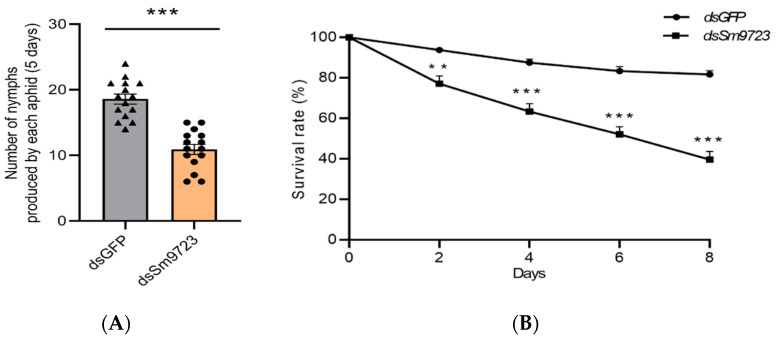
Silencing of *Sm9723* by nanocarrier-mediated RNAi reduced the number of nymphs produced by each aphid (**A**) and survival rate (**B**) of *Sitobion miscanthi*. Twelve biological replicates were conducted. Data are represented as mean ± SE. Asterisks above bars indicate significant differences between controls and treatments (** *p* < 0.01; *** *p* < 0.001, Student’s *t*-test).

As shown in Figure 7A, the number of nymphs produced by *Sm9723*-silenced aphids was significantly less than controls. In addition, Figure 7B showed that survival of *Sm9723*-silenced *S. micanthi* reduced to 63.75 ± 13.33% after feeding on wheat plants for two days, which was significantly lower than controls (dsGFP). This rate further decreased to only 39.17 ± 13.11% at eight days.

### 2.7. Effects of Sm9723 Silencing on S. miscanthi Feeding Behavior

**Figure 8:**
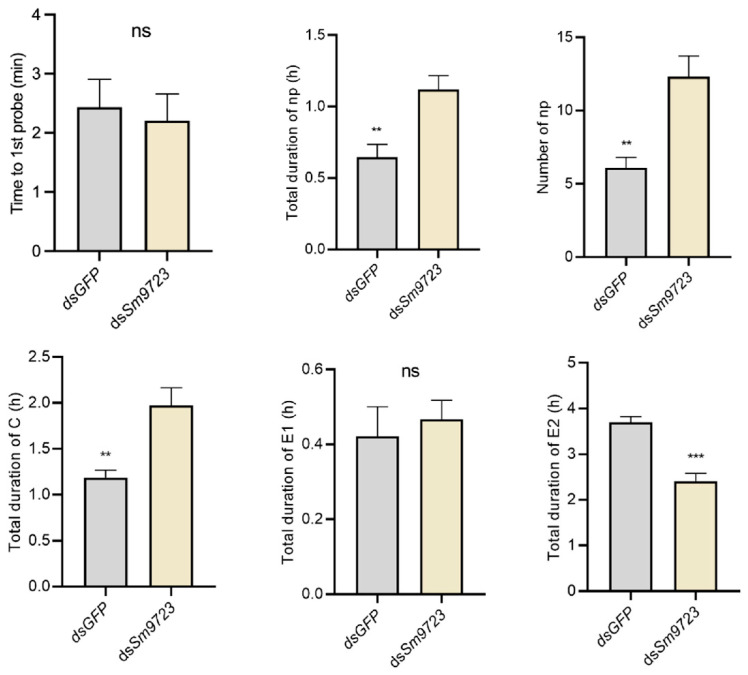
Effects of *Sm9723* silencing on feeding behavior of *Sitobion miscanthi* based on EPG recordings. Non-probing (Np); stylet probing (C); phloem salivation (E1); and phloem ingestion (E2). Data shown are mean ± SE. Asterisks above bars indicate significant differences between controls and treatments (ns, not significant; ** *p* < 0.01; *** *p* < 0.001, Mann–Whitney U test).

As shown in Figure 8, feeding behavior of *S. miscanthi* was negatively affected after inhibiting *Sm9723* expression. The time to the first probe activity of aphids treated with ds*Sm9723* was not significantly longer than that spent of the control groups. The number of non-probing (np) waveforms of *Sm9723*-silenced aphids on wheat plants was significantly greater than controls. Further, durations of the non-probing and C phases of *Sm9723*-silenced aphids feeding on wheat plants were significantly longer, but duration of phloem ingestion (E2) was significantly shorter, than aphids treated with dsGFP.

## 3. Discussion

We found that a functional uncharacterized salivary protein from *S. miscanthi* possessed a signal peptide and it was highly induced during early feeding stages of aphids on wheat plants, suggesting that Sm9723 might be involved in modulating aphid-wheat interactions.

Transient overexpression of salivary proteins in model plants, such as Arabidopsis, tobacco, and tomato, using an *Agrobacterium tumefaciens*-mediated transient transformation system is the most commonly used approach to identify aphid effectors [16,17,21]. INF1 is a PAMP from *Phytophthora* *infestans* and can trigger PTI-related PCD in *N. benthamiana* [33]. We determined that the expression of Sm9723 plus or minus signal peptide suppresses BAX- and INF1-triggered PCD in *N. benthamiana*, suggesting that Sm9723 can suppress PTI-associated PCD. A previous study showed that the signal peptide sequence is necessary for XEG1 of pathogen *Phytophthora sojae* to trigger plant defense including cell death [34]. Our results suggest that peptide signal sequence is not necessary for the involvement of Sm9723 in the suppression of plant defenses. Flg22, a PAMP derived from bacterial flagellin protein, can be recognized by transmembrane pattern-recognition receptors (PRRs) and activates callose depositions in plants [35,36]. In addition, callose deposition induced by aphid feeding is involved in sealing sieve pores as a phloem defense strategy that prevents nutrients flow to piercing-sucking insects [37]. Our results show that Sm9723 overexpression in *N. benthamiana* leaves inhibits PTI-related callose deposition.

In addition, jasmonic acid and salicylic acid are two important phytohormones conferring plant resistance against aphids [38,39]. A previous study demonstrated that the expression of the candidate effector Rp1 from the bird cherry-oat aphid *Rhopalosiphum padi* in transgenic barley lines enhanced plant susceptibility to aphids by suppressing JA and SA defense pathways [25]. We also found that the overexpression of Sm9723 in tobacco leaves suppressed the expression levels of JA and SA defense-related genes *PAL*, *PR1a*, *LOX*, and *FAD*, indicating Sm9723 is involved in suppressing plant defense as an effector. Cereal plants, such as wheat, are the host plant of *S. miscanthi*, hence the roles of Sm9723 in modulating wheat plant defense merits further investigation. However, *Agrobacterium*-mediated transformation is inefficient in cereals. An alternative approach to analyze the function of wheat aphid effectors is delivering proteins into cereal plant cells using a plant pathogen type III secretion system (T3SS), a method that has been successfully applied in the delivery of fungal effectors into wheat or barley [40]. For example, the candidate effector Pst_12806 of the fungal pathogen *Puccinia striiformis* f. sp. *tritici* can suppress PTI-associated callose deposition and defense-related gene expression in wheat when delivered by the *P. fluorescens* EtHAn delivery system [41].

Delivery of dsRNA to silence genes has also been successfully applied to analyze the roles of candidate effectors of hemipterans on host adaptability. For example, gene expression of *M. persicae* effector *MpC002* was knocked down by up to 60% after feeding on transgenic *N. benthamiana* and *A. thaliana* plants expressing dsRNA, and silenced *M. persicae* produced less nymphs, suggesting this gene is essential for aphid development on plants [42]. Salivary effector Bt56 in whiteflies *B. tabaci* was silenced by feeding with artificial diet containing dsRNA, resulting in significantly reduced whitefly performance on host plants and shorter phloem feeding durations [43]. Nanocarrier-mediated RNAi is a practical delivery system for silencing target genes of hemipteran insects, such as soybean aphid *Aphis glycines* and *M. persicae*, and is considered a promising strategy for gene function characterization and pest control [44,45,46]. In this study, to further examine the roles of *Sm9723* on *S. miscanthi* fitness, the *Sm9723* gene was silenced following application of the nanocarrier-mediated transdermal dsRNA delivery system. Our results show *Sm9723* expression levels decreased significantly after application of *dsSm9723*/nanocarriers/detergent, and its transcript levels reduced by more than 60% compared with controls, suggesting nanocarrier-mediated RNAi is an effective gene function analysis method for *S. miscanthi*.

Furthermore, we found survival and fecundity of *Sm9723*-slienced *S. miscanthi* decreased significantly, and feeding behavior of *S. miscanthi* was dramatically affected after gene silencing. For example, the non-probing phase and extracellular stylet pathway of the *Sm9723*-slienced *S. miscanthi* had a longer total duration, but duration of passive ingestion (E2 wave) of *Sm9723*-slienced *S. miscanthi* was shorter than controls, indicating aphids had trouble continuously feeding from the phloem for extensive time periods when feeding on wheat, resulting in reduced aphid performance. These outcomes suggest Sm9723 is essential for aphid adaptability to host plants as a salivary effector. Spray application of dsRNA pesticide shows great potential in controlling *M. persicae* and several plant pathogens [46,47]. Further, previous studies demonstrated that host induced gene silencing (HIGS) in transgenic wheat and barley plants targeting essential genes of *S. avenae* can successfully confer resistance against aphids [48,49]. Therefore, Sm9723 could be an ideal RNAi target gene for aphid control either through spray induced gene silencing (SIGS) or HIGS.

## 4. Conclusions

In summary, the salivary protein Sm9723 of *S. miscanthi* was shown to be involved in the suppression of plant immunity by inhibiting callose deposition, JA and SA associated defense signaling pathways. Silencing of *Sm9723* via nanocarrier-mediated RNAi resulted in significant negative effects on aphid performance and feeding behavior. The results of the current study suggested that Sm9723 plays important roles in suppressing plant defense as effector and could be potential target gene for RNAi-based aphid control.

## 5. Materials and Methods

### 5.1. Aphids and Plants

Clones of *S. miscanthi* were initially established from a single aphid collected from a wheat field without any pesticide application in Langfang city (39°51′53.21″ N, 116°61′45.96″ E), Hebei province, Northern China, which was maintained under laboratory conditions (L:D = 16 h:8 h; 20 °C ± 1 °C; 70% relative humidity) on aphid-susceptible wheat plants (*Triticum aestivum* L. Mingxian 169) for more than four years.

### 5.2. Sequence Analysis

Multiple alignment of amino acid sequences was performed using ClustalOmega (https://www.ebi.ac.uk/Tools/msa/clustalo/) (accessed on 24 April 2021). Signal peptides were predicted using the SignalP 4.1 server (http://www.cbs.dtu.dk/services/SignalP/) (accessed on 24 April 2021) and iPSORT (http://ipsort.hgc.jp/) (accessed on 24 April 2021). A phylogenetic tree was constructed by the neighbor-joining method via MEGA7.0. Bootstrap values were calculated as a percentage from over 1000 replications.

### 5.3. Agrobacterium tumefaciens Infiltration Assays to Suppress BAX/INF1-Induced Programmed Cell Death

The full coding sequence of *Sm9723* and sequence excluding signal peptide (*Sm9723^-SP^*) were cloned into pCAMBIA1300-GFP respectively, and *A. tumefaciens* GV3101 carrying pCAMBIA1300-*Sm9723-*GFP or pCAMBIA1300-*Sm9723^-SP^*-GFP constructs were infiltrated into *N. benthamiana* leaves as described in [16]. *A. tumefaciens* cells carrying BAX/INF1 were infiltrated into the same site 24 h later. Photos of leaf symptoms were taken three days after BAX/INF1 inoculation. Leaves were decolorized using ethanol, and three biological replicates were performed for each treatment. *A. tumefaciens* cells carrying GFP were used as negative controls. Primers used for gene cloning are presented in Table S1.

### 5.4. Detecting Callose Deposition in N. benthamiana Leaves

Cells of *A. tumefaciens* carrying pCAMBIA1300-GFP (control) or pCAMBIA1300-*Sm9723^-SP^-*GFP constructs were infiltrated into *N. benthamiana* leaves as described above. After 24 hpi, 20 μM Flg22 solution was injected into the same infiltration sites, and leaves were collected after 12h infiltration. Callose deposition in *N. benthamiana* leaves was visualized using aniline blue staining according to protocols reported previously [50]. Callose deposition was observed and photographed with an Echo Revolve Hybrid Microscope (Echo Laboratories, San Diego, CA, USA) using a DAPI filter. Fifteen sites (1 mm^2^/site) were selected from infiltrated areas of each treated leaf, and callose deposits was counted for each site. Three independent biological replicates were conducted.

### 5.5. RNAi Application Using the Nanocarrier-Mediated Transdermal dsRNA Delivery System

dsRNA specific for the *Sm9723* gene was synthesized and purified using a TranscriptAid T7 High Yield Transcription Kit (Thermo Fisher Scientific, Waltham, MA, USA) according to the manufacturer’s instructions using the primers listed in Table S1. The star polycation (SPc) nanocarrier was gently mixed with dsRNA at a recommended mass ratio of 1:1, and 0.05% detergent (surfactant and softened water) was added to reduce surface tension of hydrophilic nanocomplexes [44,51]. The 0.1 μL *dsSm9723*/nanocarrier/detergent and *dsGFP*/nanocarrier/detergent (Control) formulations with 200 ng/μL dsRNA were dropped on aphid notums and adsorbed immediately under the presence of detergent. Aphids were then collected at 24 or 48 h and the whole body of aphids was washed by sterilized H_2_O three times to remove nanocarrier/detergent residue and detect transcript levels of *Sm9723* following dsRNA treatment.

### 5.6. Aphid Performance

To examine the effects of *Sm9723* silencing on aphid performance, five apterous adult aphids were treated with *dsSm9723*/nanocarrier/detergent formulations, and treated aphids were transferred onto wheat plants in a plastic cage (2.5 × 2.5 × 2.5 cm) to avoid escape. The number of nymphs produced by the aphids was recorded daily for five days. Fifteen replicates were conducted for each group. In addition, ten apterous adult aphids treated with *dsSm9723*/nanocarrier/detergent were also transferred to plastic cages, and the number of surviving aphids on plants was counted daily for eight days following dsRNA treatment. Ten biological replicates were performed and aphids treated with *dsGFP*/nanocarrier/detergent were included as the control.

### 5.7. Aphid Feeding Behavior Monitoring by Electronic Penetration Graph (EPG)

Feeding behavior of the *Sm9723*-silenced adult apterous *S. miscanthi* on wheat plants was recorded using EPG (DC-EPG Giga-8, Wageningen, The Netherland). EPG monitoring was performed from 09:00 a.m. to 15:00 p.m. every day and recorded continuously for 6 h in a Faraday cage in the laboratory (20°C ± 1°C, L:D = 16:8 photoperiod, 70% RH). Each aphid and plant were used only once. The visualization and manual labelling of feeding waves were performed using Stylet^+^a. Aphid feeding waveform patterns were identified as described in [52]. Ten independent biological replicates were conducted for each treatment.

### 5.8. RT-qPCR

Total RNA of aphids and wheat leaves was extracted using TRIzol reagent according to the manufacturer’s instructions (Invitrogen, Waltham, MA, USA). 1 μg total RNA was used to synthesize the first strand cDNA using the HiScript III 1st Strand cDNA Synthesis Kit (+gDNA wiper) (Vazyme, Nanjing, China). Different aphid tissues, including the salivary gland, midgut, head, thorax and abdomen, were dissected in PBS buffer (pH 7.2) using a microscope. Expression levels of *Sm9723* in different aphid tissues were then examined using RT-qPCR analysis [31]. *β-actin* and *NADH hydrogenase* were used as internal reference genes [53]. Changes in gene expression levels involved in SA and JA defense signaling pathways, including *phenylalanine ammonia-lyase* (*PAL*)*, pathogenesis related protein-1* (*PR1a*)*, lipoxygenase* (*LOX*) and *fatty acyl desaturase* (*FAD*) of *N. benthamiana* overexpressing *Sm9723*^-SP^-GFP and GPF were also detected. RT-qPCR was performed on an ABI 7500 Real-Time PCR System (Applied Biosystems, Waltham, WA, USA) as described in [30,31]. All primers for RT-qPCR are presented in Table S1. All reactions were performed with three biological replicates, and the differential expression was calculated using the 2^–ΔΔCT^ method [54].

### 5.9. Statistical Analysis

All data were analyzed using SPSS Statistics 20.0 software (SPSS Inc., Chicago, IL, USA). Differences among groups were examined using Student’s *t*-test and one-way analysis of variance (ANOVA) test, followed by Duncan’s multiple range test. EPG data were analyzed by a Mann-Whitney U test. *p* values less than 0.05 were considered statistically significant.

## Data Availability

Not applicable.

## Supplementary Materials

The following supporting information can be downloaded at: www.mdpi.com/article/10.3390/ijms23136909/s1.
