# Supplementary material for: Salivary Effector Sm9723 of Grain Aphid Sitobion miscanthi Suppresses Plant Defense and Is Essential for Aphid Survival on Wheat
Source: Int J Mol Sci. 2022 Jun 21;23(13):6909. doi: 10.3390/ijms23136909 (PMC9266898; doi:10.3390/ijms23136909)
Supplement: Supplementary file 1 [file ijms-23-06909-s001.zip › ijms-1745021-supplementary.pdf]

Table S1. All primers used for PCR, qRT-PCR and construction.

**Primer name**	**Sequence (5'-3')**	**Purpose**
Sm9723-F	ATGAAATTGAACACATCACTT	Gene cloning of full coding sequence
Sm9723-R	CTACGCGATAGTACGTCTACGT	
Sm9723^{-SP}-F	ATTGACTTCGACGAAGAAGAGT	Gene cloning of coding sequence excluding signal peptide
Sm9723^{-SP}-R	CTACGCGATAGTACGTCTACGT	
Sm9723-q-F	ACATCCTGTTAGCCAAGGCC	RT-qPCR for aphid
Sm9723-q-R	CACCGGGTTTGTTCGAGAGA	
β-actin-F	CGTTACCAACTGGGACGATATG	
β-actin-R	GGGTTCAATGGAGCTTCTGTTA	
NADH-F	CGAGGAGAACATGCTCTTAGAC	
NADH-R	GATAGCTTGGGCTGGACATATAG	
Sm9723-F-SacI	cgagctcATGAAATTGAACACATCACTT	Transient overexpression
Sm9723-R-XbaI	gctctagaCGCGATAGTACGTCTACGT	
Sm9723^{-SP}-F-SacI	cgagctcATGATTGACTTCGACGAAGAAG	
Sm9723^{-SP}-R-XbaI	gctctagaCGCGATAGTACGTCTACGT	
dsSm9723-F	taatacgactcactataggg<br>ATTGACTTCGACGAAGAAGAGT	RNAi
dsSm9723-R	taatacgactcactataggg<br>CTACGCGATAGTACGTCTACGT	
NbPAL-F	GTTATGCTCTTAGAACGTCGCCC	RT-qPCR for *N. benthamiana*
NbPAL-R	CCGTGTAATGCCTTGTTTCTTGA	
NbPR1a-F	CGACCAGGTAGCAGCCTATG	
NbPR1a-R	TCTCAACAGCCTTAGCAGCC	
NbLOX-F	AAAACCTATGCCTCAAGAAC	
NbLOX-R	ACTGCTGCATAGGCTTTGG	
NbFAD-F	CATGTGGCTTGACTTAGTTACCTACT	
NbFAD-R	CCCTGACTTCTTTGGCTCCTT	
NbActin-F	GTTGCTATACAAGCTGTTCTCTCG	
NbActin-R	GTCAAGACGAAGAATGACATGTGG	
